# Questioning COVID-19 Surface Stability and Fomite Spreading in Three Aeromedical Cases: A Case Series

**DOI:** 10.1093/milmed/usaa548

**Published:** 2020-12-17

**Authors:** Sean Horoho, Stephen Musik, David Bryant, William Brooks, Ian M Porter

**Affiliations:** Department of Aviation Medicine, Navy Medicine Readiness and Training Unit Jacksonville, Jacksonville, FL 32212, USA; Department of Aviation Medicine, Navy Medicine Readiness and Training Unit Jacksonville, Jacksonville, FL 32212, USA; Department of Aviation Medicine, Navy Medicine Readiness and Training Unit Jacksonville, Jacksonville, FL 32212, USA; Department of Aviation Medicine, Navy Medicine Readiness and Training Unit Jacksonville, Jacksonville, FL 32212, USA; Department of Aviation Medicine, Navy Medicine Readiness and Training Unit Jacksonville, Jacksonville, FL 32212, USA

## Abstract

It is well established that coronavirus disease 2019 is primarily transmitted through respiratory droplets, and there is mounting research speculation that it may also be transmitted via fomites. Several studies have shown that the virus can persist on both porous and nonporous surfaces for hours to days, depending upon the material. This article examines three cases of polymerase chain reaction–proven severe acute respiratory syndrome coronavirus 2 infection with several additional individuals meeting CDC close contact criteria. In 1 case, 195 downstream contacts were all tested to prevent a mass outbreak in a deployment posture. Analysis of these contacts yielded only a single positive test, which could be reasonably ascribed to respiratory droplet transmission. While these cases and their contacts ultimately represent a small sample size, we suggest fomite spread may not be a significant means of transmission for severe acute respiratory syndrome coronavirus 2 in real-world operational scenarios.

## INTRODUCTION

Starting in December 2019, cases of viral pneumonia began appearing in Wuhan, Hubei Province, China. The initial cases had all been traced to the Huanan Seafood Wholesale Market, where various exotic animal meats were sold for human consumption, so it was thought that virus transmission relied upon direct contact with infected animals. Notably, on January 20, 2020, the first case of viral pneumonia was reported in Wuhan in a 35-year-old female without any known exposure to animal meat markets, wild animals, or prior cases of viral pneumonia. This infection was attributed to a novel coronavirus dubbed severe acute respiratory syndrome coronavirus (SARS-CoV)-2 that has since developed into a pandemic, coronavirus disease 2019 (COVID-19).^1–3^ The coronavirus family consists of single-stranded RNA viruses, which can infect both animals and humans, resulting in a primarily respiratory illness with multi-systemic effects. Until the emergence of SARS-CoV-2, there were six human coronaviruses identified: HCoVs-NL63, HCoVs-229E, HCoVs-OC43, HCoVs-HKU1, SARS-CoV, and Middle East respiratory syndrome-CoV.^4^

Coronavirus disease 2019 has run rampant through crowded metropolitan areas in the United States such as California and New York as well as European countries such as Spain and Italy. The exponential spread of this pandemic has resulted in never before seen travel and economic restrictions effecting an unprecedented worldwide economic contraction.^5^ As of August 28, 2020, there are 24,587,513 confirmed cases and 833,556 confirmed deaths worldwide.^6^

Since the start of this pandemic, there has been voluminous research published and our knowledge of the virus has grown, although there is still much that remains unknown. In the area of fomite spread of the virus, Doremalen et al. have shown that it can remain viable on certain surfaces ranging from hours to days. To summarize, they aerosolized SARS-CoV-2 and inoculated 50 µL droplets onto cardboard, plastic, copper, and stainless steel and incubated at 21°C to 23°C and 40% relative humidity (RH) for 7 days. During this time, virus titers were measured and it was found that there was no viable virus on cardboard after 24 hours, whereas viable virus was found on plastic, stainless steel, and copper for up to 72 hours, although titers had been significantly reduced.^7^ Chin et al. performed a similar study where 5 µL droplets of SARS-CoV-2 were deposited onto paper, wood, cloth, glass, banknote, plastic, and stainless steel and incubated at 22°C and 65% RH for 7 days. During this time, virus titers were measured and it was found that there was no viable virus on paper after 3 hours and similarly none on wood and cloth after 48 hours. Severe acute respiratory syndrome coronavirus 2 was found to be comparatively more stable on smooth, nonporous surfaces as no viable virus could be found on plastic and stainless steel after 7 days.^8^ Similarly, Biryukov et al. examined the effects of increasing temperature and humidity as it applies to fomite stability. They found that increasing temperature and RH cause the half-life of the virus to decrease across all surface types (plastic, stainless steel, and nitrile glove).^9^ Notably, these results are only applicable to indoor environments as they do not account for solar radiation, which has been shown to significantly decrease half-life.^9,10^ Reviews of fomite studies based on the severe acute respiratory syndrome and Middle East respiratory syndrome epidemics by Aboubakr et al., Kampf et al., and Ren et al. reference similar results to the persistence of SARS-CoV-2.^11–13^ It should be noted that all aforementioned studies suggest that sustained fomite transmission is possible and that they do not prove that it has occurred. Owing to the obvious moral and ethical implications with designing a study to prove this hypothesis, anecdotal case studies will likely be at the forefront to either confirm or refute this claim. It should also be noted that considerable debate exists between the terms “respiratory droplet” and “aerosol”, with both terms used interchangeably.^14^ For the purposes of this article, the term “aerosol” refers only to the experimental design of Doremalen et al. and should not be taken to imply our support or refutation of aerosols as a method of viral transmission as this is an area that requires further research.

## METHODS

We present three active duty cases from deployable squadrons aboard Naval Air Station (NAS) Jacksonville, FL, which afford multiple opportunities for fomite transmission of COVID-19 in the aviation environment. Testing for all patients was performed with polymerase chain reaction nasopharyngeal swabs. Verbal consent was obtained from all patients and their respective chain of command before the interview, and written consent was obtained after review of the complete manuscript. Only de-identified patient data have been used in this article. All close contacts were determined using the CDC criteria of within 6 feet of an infected person for at least 15 minutes starting 2 days before the symptom onset.^15^ The cases are summarized in Table I.

**TABLE I.** Summary of Case Series Demographics and Symptoms

	Case 1	Case 2	Case 3
Age/gender	22/female	20/male	19/male
Symptoms	Congestion, headache, cough, myalgia, dyspnea, and left rib pain	Headache and subjective fever	Burning sensation in chest, headache, congestion, anosmia, and ageusia
Exposure	Public restaurant	Geedunk, public beach, restaurant, and shopping center	Geedunk, public beach, restaurant, and shopping center
CDC close contacts	9	203	203
Downstream positives	1	0	0

## CASE PRESENTATIONS

### Case 1

The first case is a 22-year-old active duty female petty officer who works as a survival equipment specialist in a helicopter squadron in NAS Jacksonville, FL. She had recently returned from a shipboard deployment at sea on Friday, June 12, 2020, to Jacksonville, FL, her home duty station. The ship was in a clean bubble without any known COVID-19 infections during her entire time at sea. The following night, Saturday June 13, 2020, she went out to dinner with two of her friends from different commands at a public restaurant. Three days later (Tuesday, June 16, 2020), she started to experience congestion, which she attributed to seasonal allergies, and went to work. Over the next 2 days, she developed myalgia, headache, cough, and dyspnea and elected to stay home from work on Thursday, June 18, 2020, and Friday, June 19, 2020. On the following day, June 20, 2020, she was tested for COVID-19, which resulted positive. On June 21, 2020, she developed left rib pain and was directed to the on-base emergency department by her flight surgeon. She was evaluated for pulmonary embolus and pneumonia, which were ruled out and was discharged home in stable condition. The rest of her disease course was unremarkable, and she recovered completely.

She completed 14 days of quarantine and had a total of nine close contacts who also quarantined for 14 days and were tested for COVID-19 as per U.S. Navy policy.^16^ One of the nine close contacts tested positive on June 22, 2020, and this individual was noted to be friends with case 1 and spent time with her outside of work. The rest of the exposures were close contacts from work. During her 2 days at work while having symptoms, she handled numerous pieces of survival equipment, including a flight helmet, that were subsequently handled by others and did not lead to any downstream positive cases.

### Cases 2 and 3

Cases 2 and 3 are similar and are presented together. Case 2 is a 20-year-old active duty male attached to a fixed wing maritime patrol squadron at NAS Jacksonville, FL, and case 3 is his roommate, a 19-year-old active duty male who is attached to the same squadron. Both service members were assigned to work in the “geedunk,” a hangar convenience store, where anyone in the squadron can purchase drinks and snacks. Briefly, the geedunk is described as a 15-by-15-foot room where the attendants handle cash, credit cards, and food items and make coffee that is available for self-service. Interactions between the attendants and customers are brief and occur within approximately 3 feet.

Case 2 developed a headache and subjective fever on April 29, 2020, but did not immediately seek medical attention. Following case 3’s positive test, case 2 was tested on May 1, 2020, and experienced full symptom resolution that same day. The remainder of his clinical course was unremarkable.

Case 3 developed a burning sensation in his chest on April 30, 2020, for which he sought medical attention on May 1, 2020, and received a COVID-19 test. The burning sensation in his chest resolved within 48 hours of onset. The day following his positive test, case 3 subsequently developed a headache and congestion that resolved on day 4 and coincided with the development of anosmia and ageusia, which persisted for 1 month with gradual improvement but without complete resolution. The remainder of case 3’s clinical course was unremarkable except that he remained in quarantine for 21 days because of persistent anosmia and ageusia.

During the 48 hours preceding the appearance of symptoms, both patients worked as cashiers in the geedunk and spent time at the local beach, restaurants, and a shopping center. Eight individuals met the CDC close contact criteria for testing and an additional 195 personnel were tested twice over the course of 3-7 days because of concern for exposure in the geedunk while in a deployment status. Despite the large number of tested individuals, no new positive cases were identified and the affected squadrons deployed successfully without any downstream positives.

## DISCUSSION

Based on health surveillance data, Duval County, the home of NAS Jacksonville and one of the major metropolitan areas in Florida, ranks fourth among all of Florida’s metropolitan areas in terms of the total number of positive COVID-19 cases dating back to the start of the pandemic. It is surpassed only by Miami, Tampa/St. Petersburg, and Orlando.^17^ For reasons of operational security, the Navy does not report the total number of cases aboard an installation; however, both the city of Jacksonville and NAS Jacksonville did see a dramatic uptick in positive cases in June 2020.^18^ These positive cases presented their own set of unique aeromedical challenges, which forced local medical assets to deal with protecting operational forces while maximizing decreased risk of transmission. Real-world operational examples of spreading COVID-19 in similar environments are seen with the aircraft carrier USS Theodore Roosevelt and destroyer USS Kidd where 1,102 and 78 confirmed cases respectively were reported giving plenty of concern for aeromedical assets given their breadth of travel.^19^

Although respiratory droplet transmission has been shown to be the primary method of transmission of SARS-CoV-2, multiple studies have shown that it remains viable on both porous and nonporous surfaces for time periods ranging from hours to days. Although these studies were not designed to definitively prove fomite transmission, they do suggest that it is a possibility. The three cases described above further highlight the potency of droplet spread and its role in spreading COVID-19.^14^ Indeed, most if not all cases met the CDC close contact criteria of closer than 6 feet for at least 15 minutes.^15^ Furthermore, Doremalen et al. confirmed that the half-life of SARS-CoV-2 in aerosols was similar to that of SARS-CoV-1, with median estimates of approximately 1 hour.^9^ Combining this research with the anecdotal evidence previously presented, it is quite plausible that droplet transmission was responsible for these cases as case 1’s downstream positive was a friend and cases 2 and 3 were roommates. Furthermore, inconsistent compliance with face coverings could have also played a role in transmission between cases and downstream positives.

Conversely, the previously hypothesized fomite transmission of SARS-CoV-2 is not supported in this case series data. In the first case, of nine close contacts, only one tested positive and this was likely due to prolonged exposure from outside of work. Interactions without a proper facial covering increase the spread of COVID-19, whereas the proper wearing of a facial covering has a significant effect on reducing virus transmission.^20^ Importantly, there were no documented exposures from the confirmed positive case who handled many pieces of critical equipment, to include fitting a flight helmet. In the second and third cases, there were 195 potential exposures who may have shared multiple surfaces to include counters, cash, credit cards, and food wrappers, and all tested negative twice in a period of 3-7 days. If fomite spread is as important a method of transmission for SARS-CoV-2 as previously theorized, then it is reasonable to expect that of the 200+ potential contacts that there would have been some number of positive cases resulting from commonly stored or touched items. Since this was not the case, this leads the authors to question whether or not fomite spread plays as important a role as suggested by in vitro research as compared to other common respiratory pathogens.

Vulnerabilities in this data include potential over reporting of being a close contact because of different motivations such as fear of stopping movement, as a good proportion of the 195 individuals tested were set to deploy. In addition, this is an observational descriptive study where controlling for external variables was not possible. False negatives from polymerase chain reaction testing are also a possibility but refuted by repeat testing in one portion of the data. This was a result of local medical assets employing their best judgment on how to ensure zero downstream positives by requiring two negative tests before deploying or embarking on aircraft. During the time of these cases, social distancing, masking, and sanitizing were in practice and may lend further support to their continued use and value.

## CONCLUSION

The cases of viral pneumonia that started in Wuhan, China in December 2019 quickly swept the globe and evolved into the COVID-19 pandemic. Since that time, much research has been conducted on the epidemiology and pathobiology of the virus, such that there is now confirmed respiratory droplet transmission and proposed fomite transmission. Although there is reliable evidence that shows the ability of the virus to persist on both porous and nonporous surfaces for hours to days, there is no evidence which shows that this has actually resulted in a human case of COVID-19. In this case series, we have instead demonstrated that fomite spread may not be as large of a factor in transmission as was originally thought, when compared to other common respiratory illnesses. Although the authors realize that a small anecdotal case series does not equate to disproving the role of fomite spread in the transmission of SARS-CoV-2, it is our hope that this furthers the conversation and continues to encourage continued operational posture while employing proven practices such as social distancing, masking, and sanitizing. It is our belief that there is a need for further experimental research to determine the role, if any, that fomite spread plays in the transmission of SARS-CoV-2 compared with other similar type respiratory illnesses. We further suggest other case series research as ethical limitations will continue to challenge answering these questions.
